# Cigar smoking prevalence and morbidity among US adults, 2000–2015

**DOI:** 10.1016/j.pmedr.2019.100821

**Published:** 2019-02-11

**Authors:** Brian L. Rostron, Catherine G. Corey, Renee M. Gindi

**Affiliations:** a Center for Tobacco Products, Food and Drug Administration, Beltsville, MD, United States of America; b National Center for Health Statistics, Centers for Disease Control and Prevention, Hyattsville, MD, United States of America

**Keywords:** Cigar, Tobacco, Cardiovascular, Stroke, Cancer

## Abstract

Cigar smoking causes many of the same health conditions as cigarettes, but less information is available on prevalence of use trends and the disease burden of cigar smoking in the US. To examine these issues, we analyzed cigar use and health condition data from the National Health Interview Survey from 2000, 2005, 2010, and 2015, estimating prevalence of use by year and over time. We also estimated the number of, and adjusted disease prevalence ratios for, US adults aged ≥35 years with self-reported history of heart disease, stroke, or cancer attributable to cigar smoking. We found that prevalence of current cigar smoking has remained generally stable at around 2.3% among US adults aged ≥18 years between 2000 and 2015 but has increased among female and non-Hispanic black adults. Former exclusive cigar smokers were more likely to report having had heart conditions (aPR = 1.33, 95% CI = 1.03–1.72), stroke (aPR = 2.42, 95% CI = 1.57–3.75), and cancer (aPR = 1.44, 95% CI = 1.09–1.88) than never cigar smokers. It is estimated that nearly 200,000 cardiovascular conditions and cancer cases among US adults are attributable to former exclusive cigar smoking. This analysis shows that prevalence of current cigar smoking has remained stable among US adults but has increased among certain demographic groups. Former exclusive cigar use is associated with increased prevalence of heart disease, stroke, and cancer, which may result in part from smoking cessation following disease onset.

## 1 Introduction

Cigar smoking causes many of the same health conditions as cigarette smoking (Centers for Disease Control and Prevention, 2018; National Cancer Institute, 1998), including oral cancer, lung cancer, and heart disease (Chang et al., 2015). It has also been estimated that regular cigar use is responsible for approximately 9000 deaths in the US each year, which represent a loss of 140,000 life-years (Nonnemaker et al., 2014). Even so, much less information is available on trends in cigar smoking prevalence and the disease burden of cigar use. Some large national surveys, such as the National Survey on Drug Use and Health (Substance Abuse and Mental Health Services Administration, 2018) and the Tobacco Use Supplement to the Current Population Survey (National Cancer Institute, 2018), collect information on cigar smoking but do not use a minimum lifetime threshold for cigars smoked and thus may overestimate the prevalence of established cigar smokers (Ryan et al., 2012). In contrast, the National Health Interview Survey (NHIS) has consistently included a lifetime threshold for cigars smoked, allowing for classification based on cumulative exposure. NHIS also contains information on health behaviors and conditions, allowing for estimation of the disease burden of cigar use, similar to previously-published estimates of cigarette smoking-attributable morbidity (Rostron et al., 2014).

Using data from the 2000, 2005, 2010, and 2015 NHIS, we estimated US cigar smoking prevalence by year and over time among all adults. We also analyzed the association between cigar use and self-reported medical conditions, including heart conditions, stroke, and cancer, and calculated adjusted disease prevalence ratios (aPRs) for exclusive cigar use among adults ≥35 years. We then used these aPRs to estimate the number of cardiovascular conditions and cancer cases attributable to former exclusive cigar smoking among US adults aged ≥35.

## 2 Methods

NHIS is a nationally representative cross-sectional survey of the US civilian noninstitutionalized population. Sampled adults were asked about cigar use in the 2000, 2005, 2010, and 2015 Cancer Control Supplements; response rates to the Sample Adult module ranged from 77.3% (2010) to 82.6% (2000) and there were approximately 30,000 respondents each year. We calculated cigar smoking prevalence for adults aged ≥18 years overall and by sex, race/ethnicity, and age group. Current established cigar use was defined as having ever smoked ≥50 cigars and currently smoking every day or some days, former established use as having smoked ≥50 cigars and currently not smoking at all, and never use as having smoked <50 cigars (Office of Disease Prevention and Health Promotion, 2014). We also calculated exclusive cigar smoking prevalence for adults aged ≥35 years, defined as cigar users who reported not having ever: smoked ≥100 cigarettes; used either chewing tobacco, snuff, or smokeless tobacco ≥20 times; smoked a pipe ≥50 times (2000, 2005); smoked bidis ≥20 times (2000, 2005); or used a traditional pipe, waterpipe, or e-cigarette (2015). Use of cigars in conjunction with other tobacco products was also estimated. Changes over time were assessed using logistic regression.

Respondents reported if they had ever been told by a health professional that they had a heart condition (angina, coronary heart disease, heart attack, or other heart disease), stroke, or cancer. In a pooled analysis using all years of data, we used a marginal predictions approach with logistic regression (Bieler et al., 2010) to calculate aPRs for having been diagnosed with a medical condition by cigar smoking status. The aPRs were adjusted for sex, age, race/ethnicity, educational attainment, alcohol consumption, and body mass index. We estimated disease prevalence ratios only among adults aged ≥35 years to allow for cigar use to produce health effects, consistent with other estimates of cigarette smoking-attributable morbidity (Rostron et al., 2014) and mortality (Centers for Disease Control and Prevention, 2008). To isolate the effects of cigar smoking, we restricted this analysis to exclusive cigar users. For conditions with statistically-significant (*p*-value < 0.05) aPRs, we calculated the number of US adults in 2015 with cigar smoking-attributable medical conditions as *N* × *P*_*s*_ × *P*_*d*_|_*ns*_ × (*aPR* − 1) (Rostron et al., 2014), where *N* is adults aged ≥35 years in 2015 (US Census Bureau, 2017), *P*_*s*_ is former exclusive cigar smoking prevalence (2000, 2005, 2010, 2015), and *P*_*d*_|_*ns*_ is history of diagnosed disease among never cigar smokers (2000, 2005, 2010, 2015). A spreadsheet presenting all data inputs and calculations and a technical note explaining the methodology in greater detail are available as Supplementary material.

## 3 Results

Current cigar smoking prevalence among US adults overall was generally consistent from 2000 to 2015 at around 2.3%, but increased among some subgroups, including females (0.2% to 0.6%) and non-Hispanic black adults (2.0% to 3.3%) (Table 1). The prevalence of former cigar smoking also increased among females (0.3% to 0.8%) and adults aged 25–34 years (1.7% to 3.5%), and decreased among those aged ≥65 years (8.1% to 6.6%) and adults of other non-Hispanic race/ethnicity (3.4% to 1.4%). Use of cigarettes and other tobacco products was common among cigar smokers (Supplemental Table 1). Prevalence of current and former exclusive cigar smoking among adults aged ≥35 years was generally stable during this time, although there was a non-significant decline in current exclusive cigar smoking from 2000 to 2015 (coefficient for trend = −0.09, *p* = 0.08). (Additional prevalence information for exclusive cigar smokers is available in Supplemental Table 2). After excluding users of all other tobacco products from the analysis, aPRs for former exclusive cigar smokers compared to never cigar smokers were elevated for heart conditions (aPR = 1.33, 95% CI = 1.03–1.72), stroke (aPR = 2.42, 95% CI = 1.57–3.75), and all cancers (aPR = 1.44, 95% CI = 1.09–1.88), although estimates were not elevated for current exclusive cigar smokers (Table 2). In 2015, an estimated 65,000 heart conditions, 62,000 strokes, and 66,000 cancer cases among US adults aged ≥35 years were attributable to former exclusive cigar smoking.

**Table 1:** Trends in prevalence of US adult cigar smoking, NHIS 2000–2015.

	2000	2005	2010	2015	*p* for trend
	Weighted % (95% CI)	Weighted % (95% CI)	Weighted % (95% CI)	Weighted % (95% CI)	
Current cigar smoking, aged ≥ 18 years^a^					
Overall	2.3 (2.1–2.5)	2.2 (2.0–2.5)	2.5 (2.3–2.8)	2.3 (2.0–2.5)	0.62
Sex					
Male	4.5 (4.1–4.9)	4.3 (3.9–4.8)	4.7 (4.2–5.2)	4.1 (3.7–4.6)	0.39
Female	0.2 (0.1–0.3)	0.3 (0.2–0.4)	0.5 (0.4–0.6)	0.6 (0.4–0.7)	<**0.01**
Age group (years)					
18–24	2.1 (1.5–2.8)	2.2 (1.6–3.0)	2.3 (1.7–3.2)	2.3 (1.7–3.2)	0.53
25–34	2.2 (1.8–2.7)	2.3 (1.8–2.8)	2.6 (2.1–3.3)	2.8 (2.2–3.5)	0.09
35–64	2.7 (2.4–3.0)	2.7 (2.4–2.9)	2.8 (2.5–3.2)	2.4 (2.1–2.8)	0.34
65+	1.1 (0.8–1.5)	0.9 (0.7–1.3)	1.5 (1.2–2.1)	1.3 (1.0–1.7)	0.10
Race/ethnicity					
Non-Hispanic white	2.5 (2.2–2.7)	2.6 (2.3–2.9)	2.8 (2.5–3.2)	2.4 (2.1–2.8)	0.86
Non-Hispanic black	2.0 (1.6–2.5)	1.5 (1.1–1.9)	2.7 (2.2–3.3)	3.3 (2.7–4.1)	<**0.01**
Hispanic	1.5 (1.1–2.0)	1.6 (1.2–2.1)	1.4 (1.0–2.0)	1.3 (1.0–1.8)	0.51
Non-Hispanic other	1.6 (1.0–2.4)	0.8 (0.4–1.5)	1.5 (1.0–2.2)	1.2 (0.8–1.8)	0.74

Former cigar smoking, aged ≥ 18 years^b^					
Overall	4.3 (4.0–4.6)	4.6 (4.4–4.9)	4.7 (4.4–5.0)	4.3 (3.9–4.6)	0.80
Sex					
Male	8.7 (8.2–9.3)	9.3 (8.8–9.9)	9.0 (8.4–9.6)	8.0 (7.4–8.7)	0.06
Female	0.3 (0.2–0.3)	0.4 (0.3–0.5)	0.7 (0.5–0.8)	0.8 (0.6–1.0)	<**0.01**
Age group (years)					
18–24	1.7 (1.3–2.3)	1.9 (1.4–2.5)	1.6 (1.1–2.4)	2.0 (1.4–2.8)	0.77
25–34	1.7 (1.4–2.2)	2.1 (1.7–2.6)	2.9 (2.4–3.5)	3.5 (2.9–4.3)	<**0.01**
35–64	4.7 (4.3–5.1)	4.9 (4.5–5.4)	4.8 (4.3–5.2)	4.2 (3.8–4.7)	0.07
65+	8.1 (7.2–9.0)	8.8 (8.0–9.6)	8.9 (8.0–9.9)	6.6 (5.8–7.4)	**0.01**
Race/ethnicity					
Non-Hispanic white	5.0 (4.7–5.4)	5.7 (5.3–6.1)	5.8 (5.4–6.2)	5.4 (5.0–5.9)	0.17
Non-Hispanic black	2.3 (1.7–2.9)	2.1 (1.7–2.7)	2.7 (2.2–3.2)	3.0 (2.4–3.8)	0.05
Hispanic	1.8 (1.3–2.4)	1.7 (1.3–2.2)	2.3 (1.8–2.8)	1.7 (1.3–2.2)	0.95
Non-Hispanic other	3.4 (2.5–4.6)	3.0 (2.1–4.2)	2.2 (1.5–3.2)	1.4 (1.0–2.0)	<**0.01**

Exclusive cigar smoking, aged ≥ 35 years^c^					
Current cigar smoking	0.8 (0.7–1.1)	0.9 (0.8–1.2)	1.2 (0.9–1.6)	0.5 (0.4–0.7)	0.08
Former cigar smoking	0.8 (0.6–1.0)	0.9 (0.7–1.1)	1.3 (1.1–1.6)	0.8 (0.6–1.0)	0.81

a Current cigar smoking refers to those who reported smoking ≥50 cigars during their lifetime and now smoking cigars every day or some days.

b Former cigar smoking refers to those who reported smoking ≥50 cigars during their lifetime and now smoking cigars not at all.

c Exclusive cigar smoking refers to those cigar smokers who reported not having ever smoked ≥100 cigarettes; used either chewing tobacco, snuff, or smokeless tobacco ≥20 times; smoked a pipe ≥50 times (2000, 2005); smoked bidis ≥20 times (2000, 2005), or used a traditional pipe, water pipe, or e-cigarette (2015).

**Table 2:** Adjusted prevalence ratios and morbidity burden by disease and cigar smoking status aged ≥35 years,^a^ 2000, 2005, 2010, and 2015 NHIS.

Disease	Adjusted prevalence ratio^b^ in 2000–2015 (95% CI)		Cigar smoking-attributable cases among former exclusive cigar smokers in 2015 (95% CI)
	Current exclusive cigar smoker	Former exclusive cigar smoker	
Heart conditions^c^	0.88 (0.61–1.27)	1.33 (1.03–1.72)	65,000 (46,000–93,000)
Stroke	0.88 (0.39–1.99)	2.42 (1.57–3.75)	62,000 (21,000–180,000)
All cancer	0.83 (0.51–1.35)	1.44 (1.09–1.88)	66,000 (44,000–99,000)

a Current cigar smoking was defined as having ever smoked ≥50 cigars and now smoking cigar every day or some days, former cigar smoking as having smoked ≥50 cigars and now not smoking cigars at all, and never cigar smoking (the reference group) as not having smoked ≥50 cigars. Individuals were excluded from this analysis if they reported having smoked at least 100 cigarettes; used either chewing tobacco, snuff, or smokeless tobacco ≥20 times; smoked a pipe ≥50 times (2000, 2005); smoked bidis ≥20 times (2000, 2005), or ever used a traditional pipe, water pipe, or e-cigarette (2015).

b Prevalence ratios were adjusted for sex, age, race/ethnicity, educational attainment, alcohol consumption, and body mass index and estimated using NHIS sampling weights to account for the complex survey design.

c Heart conditions include angina, coronary heart disease, heart attack and other heart disease.

## 4 Discussion

US adult current cigar smoking prevalence was relatively stable from 2000 to 2015 at around 2.3%. These results are consistent with previously-reported trends using more limited data (Agaku and Alpert, 2016); however, they characterize the trends among established cigar smokers for a longer period. Statistically significant increases in current cigar smoking were seen among female and non-Hispanic black adults, which coincided with changes in cigar flavor availability and pack sizes and price, particularly in the mass-merchandise market (Delnevo et al., 2017). A small increase in point estimates for cigar smoking prevalence in 2010 may have been due to changes in federal taxation of tobacco products, (Agaku and Alpert, 2016) although cigar smoking prevalence was generally stable during the extended period.

Cigar smoking was significantly associated with prevalence of heart conditions, stroke, and cancer, although for former cigar smokers only. This result is consistent with previous findings for mortality risks associated with current cigar smoking (Chang et al., 2015; Nonnemaker et al., 2014), although it adds to the research literature by identifying increased prevalence of stroke, in addition to heart disease, among former cigar users compared to never users. The observed increased disease prevalence among former but not current cigar smokers in this study may reflect in part smoking cessation following disease onset.

This study has certain limitations. Smoking status and medical conditions were self-reported by survey participants and may be underreported or underdiagnosed, and the extent of misclassification could change over time. Self-reported conditions at time of interview represent the population prevalence of having ever been diagnosed with the condition, not the prevalence or incidence of the condition. Due to sample size considerations, we analyzed all cancers as an outcome, rather than specific cancers. This heterogeneity could bias the association observed towards the null. Prevalence ratios were adjusted for important demographic characteristics and health risk factors such as sex, race/ethnicity, and alcohol consumption, but this does not include all potential confounders. All covariates were measured at the time of interview, and associations between potential confounders and outcomes studied may have been attenuated due to behavioral changes following disease onset, a limitation inherent in using cross-sectional data. NHIS only asked about bidis, pipes, and e-cigarettes in certain years, but sensitivity analyses indicated that the effect of inclusion of these products on prevalence ratios was minimal. NHIS did not ask cigar smokers about the types or quantities of cigars that they used. Cigar type may influence frequency of use and depth of inhalation, which can affect the disease risks of cigar smoking. It is not known to what extent different cigar types contribute to the observed increased disease prevalence and burden for former cigar smokers. NHIS also did not ask former cigar smokers about the timing of their cigar smoking cessation.

## 5 Conclusion

This analysis has shown that nearly 200,000 cardiovascular conditions and cancer cases were attributable to exclusive cigar smoking among US adults aged ≥35 years in 2015. These results are consistent with previous findings that cigar smoking is associated with preventable diseases.

## Disclaimer

The findings and conclusions in this publication are those of the authors and do not represent the official position or policy of the National Center for Health Statistics, Centers for Disease Control and Prevention and Center for Tobacco Products, Food and Drug Administration.

## Conflict of interest statement

The authors have no conflicts of interest to declare.

## Appendix A Supplementary data

Supplementary tables Image 1

Supplementary material 1 Image 2

Supplementary material 2 Image 3

## References

[bb0005] Agaku I.T., Alpert H.R. (2016). Trends in annual sales and current use of cigarettes, cigars, roll-your-own tobacco, pipes, and smokeless tobacco among US adults, 2002–2012. Tob. Control..

[bb0010] Bieler G.S., Brown G.G., Williams R.L., Brogan D.J. (2010). Estimating model-adjusted risks, risk differences, and risk ratios from complex survey data. Am. J. Epidemiol..

[bb0015] Centers for Disease Control and Prevention (2008). Smoking-attributable mortality, years of potential life lost, and productivity losses—United States, 2000–2004. Morb. Mortal. Wkly Rep..

[bb0020] Centers for Disease Control and Prevention (2018). Cigars Fact Sheet. https://www.cdc.gov/tobacco/data_statistics/fact_sheets/tobacco_industry/cigars/.

[bb0025] Chang C.M., Corey C.G., Rostron B.L., Apelberg B.J. (2015). Systematic review of cigar smoking and all cause and smoking related mortality. BMC Public Health.

[bb0030] Delnevo C.D., Giovenco D.P., Miller Lo E.J. (2017). Changes in the mass-merchandise cigar market since the tobacco control act. Tob. Regul. Sci..

[bb0035] National Cancer Institute (1998). Cigars: health effects and trends.

[bb0040] National Cancer Institute (2018). Tobacco use supplement to the current population survey. https://cancercontrol.cancer.gov/brp/tcrb/tus-cps/.

[bb0045] Nonnemaker J., Rostron B., Hall P., MacMonegle A., Apelberg B. (2014). Mortality and economic costs from regular cigar use in the United States, 2010. Am. J. Public Health.

[bb0050] Office of Disease Prevention and Health Promotion (2014). Healthy People 2020 - reduce use of cigars, cigarillos, and little filtered cigars by adults. https://www.healthypeople.gov/node/5289/data_details.

[bb0055] Rostron B.L., Chang C.M., Pechacek T.F. (2014). Estimation of cigarette smoking-attributable morbidity in the United States. JAMA Intern. Med..

[bb0060] Ryan H., Trosclair A., Gfroerer J. (2012). Adult current smoking: differences in definitions and prevalence estimates—NHIS and NSDUH, 2008. J. Environ. Public Health.

[bb0065] Substance Abuse and Mental Health Services Administration (2018). National survey of drug use and health. https://www.samhsa.gov/data/population-data-nsduh.

[bb0070] US Census Bureau (2017). Annual estimate of the resident population for the United States, July 1, 2015. https://www.census.gov/data/tables/2016/demo/popest/nation-detail.html.

