# Supplementary material for: Cigar smoking prevalence and morbidity among US adults, 2000–2015
Source: Prev Med Rep. 2019 Feb 11;14:100821. doi: 10.1016/j.pmedr.2019.100821 (PMC6378850; doi:10.1016/j.pmedr.2019.100821)
Supplement: Supplementary file 1 — Supplementary tables [file mmc1.pdf]

Supplemental Table 1. Prevalence of Cigarette and Other Tobacco Product Use, Among US Adult Current and Former Cigar Smokers Aged >=18 years, NHIS 2000-2015

	2000	2005	2010	2015	
	**Weighted % (95%CI)**	**Weighted % (95%CI)**	**Weighted % (95%CI)**	**Weighted % (95%CI)**	***p* for trend**
**Current cigar smoking***					
Current cigarette smoking#	47.7 (43.1-52.3)	47.3 (42.7-52.0)	45.8 (40.8-50.9)	40.4 (35.3-45.8)	**0.03**
Current use of other tobacco products$	16.1 (12.7-20.1)	16.5 (13.3-20.3)	10.5 (7.8-14.1)	27.5 (22.7-32.9)	**<0.01**
**Former cigar smoking^**					
Current cigarette smoking#	28.1 (25.4-31.0)	28.7 (25.8-31.8)	25.9 (23.1-28.9)	29.5 (26.0-33.3)	0.84
Current use of other tobacco products$	8.0 (6.5-9.8)	9.8 (8.2-11.7)	7.8 (6.2-9.8)	19.5 (16.5-22.9)	**<0.01**

* Current cigar smoking refers to those who reported smoking ≥50 cigars during their lifetime and now smoking cigars every day or some days.

^ Former cigar smoking refers to those who reported smoking ≥50 cigars during their lifetime and now smoking cigars not at all.

Current cigarette smoking includes reported having smoked at least 100 cigarettes and smoking every day or some days at the time of the survey.

$ Other tobacco products includes using chewing tobacco, snuff, or smokeless tobacco ≥20 times and using every day or some days at the time of the survey, or smoking a pipe ≥50 times and using every day or some days at the time of the survey (2000, 2005), or smoking bidis ≥20 times and using every day or some days at the time of the survey (2000, 2005), or using a traditional pipe, water pipe, or e-cigarette and using every day or some days at the time of the survey (2015).

Supplemental Table 2. Trends in Prevalence of Exclusive Cigar Smoking, US Adults Aged ≥ 35 years, NHIS 2000-2015

	2000	2005	2010	2015	
	**Weighted % (95%CI)**	**Weighted % (95%CI)**	**Weighted % (95%CI)**	**Weighted % (95%CI)**	***p* for trend**
**Current exclusive cigar smoking***					
Overall	0.8 (0.7-1.1)	0.9 (0.8-1.2)	1.2 (0.9-1.6)	0.5 (0.4-0.7)	0.08
Sex					
Male	2.2 (1.7-2.9)	2.3 (1.9-2.9)	3.1 (2.4-4.0)	1.3 (0.9-1.8)	**0.02**
Female	0.0 (0.0-0.1)#	0.1 (0.0-0.2)#	0.0 (0.0-0.1)#	0.1 (0.0-0.2)#	0.84
Age group (years)					
35-64	1.0 (0.7-1.3)	1.1 (0.9-1.4)	1.3 (1.0-1.7)	0.7 (0.5-1.0)	0.16
65+	0.4 (0.2-0.9)#	0.4 (0.2-0.7)#	1.0 (0.6-1.7)	0.1 (0.0-0.2)#	0.17
Race/ethnicity					
Non-Hispanic white	1.0 (0.7-1.3)	1.1 (0.8-1.4)	1.5 (1.1-2.0)	0.5 (0.3-0.8)	0.13
Non-Hispanic black	0.9 (0.5-1.6)#	0.8 (0.4-1.3)	0.9 (0.5-1.6)	1.1 (0.6-2.0)	0.39
Hispanic	0.4 (0.1-1.0)#	0.8 (0.4-1.7)#	0.9 (0.5-1.7)#	0.4 (0.2-0.8)#	0.64
Non-Hispanic other	0.2 (0.0-0.8)#	0.2 (0.0-0.9)#	0.2 (0.0-0.8)#	0.1 (0.0-0.5)	0.37
**Former exclusive cigar smoking^**					
Overall	0.8 (0.6-1.0)	0.9 (0.7-1.1)	1.3 (1.1-1.6)	0.8 (0.6-1.0)	0.81
Sex					
Male	2.1 (1.7-2.7)	2.2 (1.8-2.8)	3.1 (2.5-3.8)	1,8 (1.4-2.5)	0.78
Female	0.0 (0.0-0.1)#	0.1 (0.0-0.2)#	0.2 (0.1-0.3)	0.0 (0.0-0.1)#	0.37
Age group (years)					
35-64	0.7 (0.5-0.9)	0.8 (0.6-1.0)	1.0 (0.8-1.3)	0.8 (0.6-1.1)	0.29
65+	1.3 (0.9-1.9)	1.3 (0.9-1.9)	2.1 (1.6-2.9)	0.7 (0.4-1.3)#	0.21
Race/ethnicity					
Non-Hispanic white	0.9 (0.7-1.2)	1.0 (0.8-1.3)	1.6 (1.2-2.0)	0.9 (0.6-1.3)	0.40
Non-Hispanic black	0.3 (0.1-0.9)#	0.6 (0.3-1.2)#	1.0 (0.5-1.7)	0.9 (0.4-1.8)#	0.09
Hispanic	0.8 (0.4-1.4)#	0.8 (0.4-1.5)#	0.7 (0.4-1.1)	0.4 (0.2-0.8)#	0.08
Non-Hispanic other	0.4 (0.1-1.7)#	0.3 (0.1-1.4)#	0.6 (0.2-1.3)#	0.1 (0.0-0.9)#	0.37

* Exclusive current cigar smoking refers to those who reported smoking ≥50 cigars during their lifetime who now smoking cigars every day or some days and who reported not having ever smoked ≥100 cigarettes; used either chewing tobacco, snuff, or smokeless tobacco ≥20 times; smoked a pipe ≥50 times (2000, 2005); smoked bidis ≥20 times (2000, 2005), or used a traditional pipe, water pipe, or e-cigarette (2015).

^ Former cigar smoking refers to those who reported smoking ≥50 cigars during their lifetime who now smoking cigars not at all and who reported not having ever smoked ≥100 cigarettes; used either chewing tobacco, snuff, or smokeless tobacco ≥20 times; smoked a pipe ≥50 times (2000, 2005); smoked bidis ≥20 times (2000, 2005), or used a traditional pipe, water pipe, or e-cigarette (2015).

Relative standard error (RSE) > 30%.
