# Supplementary material for: Cigar smoking prevalence and morbidity among US adults, 2000–2015
Source: Prev Med Rep. 2019 Feb 11;14:100821. doi: 10.1016/j.pmedr.2019.100821 (PMC6378850; doi:10.1016/j.pmedr.2019.100821)
Supplement: Supplementary file 2 — Supplementary material 1 [file mmc2.pdf]

# Calculation of Cigar Smoking-Attributable Morbidity and Variance Estimates

Smoking-attributable morbidity, $SAM_b$, is calculated as the number of persons who have had a particular medical condition due to smoking using the following formula:

$$SAM_b = N * P_e * P_{d|ns} * (RR - 1)$$

where $N$ is the number of persons in the population, $P_e$ is prevalence of the smoking status, $P_{d|ns}$ is the proportion of never smokers who have had the condition, and $RR$ is relative risk of the condition by smoking status.

The variance of $SAM_b$ was originally calculated as the product of three independent random variables with $N$ treated as a constant in the following manner:

$$\begin{aligned} Var(SAM_b) \quad = \quad & N^2 * (P_e^2 P_{d|ns}^2 Var(RR-1) + P_e^2 (RR-1)^2 Var(P_{d|ns}) + \\ & P_{d|ns}^2 (RR-1)^2 Var(P_e) + P_e^2 Var(P_{d|ns}) Var(RR-1) + \\ & P_{d|ns}^2 Var(P_e) Var(RR-1) + (RR-1)^2 Var(P_e) Var(P_{d|ns}) + \\ & Var(P_e) Var(P_{d|ns}) Var(RR-1)) \end{aligned}$$

$Var(RR-1)$ is estimated as $Var(RR)$ using the delta method as a Taylor series where $Var(f(x)) \approx (f'x)^2 Var(x)$. $Var(RR)$ is estimated as $Var(e^x)$ where $x$ is a random variable with mean $u$ equal to log $RR$ such that $Var(e^x) \approx (e^x)^2 Var(x)$ and $Var(RR) \approx RR^2 Var(logRR)$.

It has been suggested that this approach may be excessively conservative and that the variance of the product of the three random variables, which is generally bounded by 0 and 1, could be estimated on the logarithmic scale. In this case,

$$\begin{aligned} Var(log(SAM_b)) &= Var(log(N) + log(Pe) + log(Pd) + log(RR-1)) \\ &= Var(log(Pe)) + Var(log(Pd)) + Var(log(RR-1)) \end{aligned}$$

$Var(log(RR-1))$ is estimated as $Var(log(RR))$ and $Var(log(Pe))$ and $Var(log(Pd))$ are again estimated using the delta method where $Var(f(x)) \approx (f'x)^2 Var(x)$. In this case, $Var(f(x)) \approx (1/x)^2 Var(x)$ or $Var(log(P)) \approx \frac{Var(P)}{P^2}$. Confidence intervals for $SAM_b$ are constructed by exponentiating the standard error of $log(SAM_b)$.
