# Supplementary material for: Cigar smoking prevalence and morbidity among US adults, 2000–2015
Source: Prev Med Rep. 2019 Feb 11;14:100821. doi: 10.1016/j.pmedr.2019.100821 (PMC6378850; doi:10.1016/j.pmedr.2019.100821)
Supplement: Supplementary file 3 — Supplementary material 2 [file mmc3.pdf]

Appendix to Cigar Smoking Prevalence and Morbidity Among US Adults, 2000-2015

Medical condition	Cigar smoking status	Population Size	Cigar Smoking Prevalence			Disease Prevalence among Never Smokers		
		N - US population size 35+	Pe - prevalence of exclusive cigar smoking 35+	Variance(Pe)	Var(log(Pe))	P d|ns - prevalence of disease among never smokers 35+	Variance(P d|ns)	Var(log(P d|ns))
Heart conditions*	Former Exclusive	172100973	0.0094	0.00000036	0.004074242	0.1221	0.0000040	0.0002683
Stroke	Former Exclusive	172100973	0.0094	0.00000036	0.004074242	0.0270	0.0000008	0.0011111
Any cancer	Former Exclusive	172100973	0.0094	0.00000036	0.004074242	0.0924	0.0000032	0.0003795
Sum of conditions								

*Heart conditions includes angina, coronary heart disease, heart attack, and other heart disease

^RR is estimated by the adjusted prevalence ratio.

Disease Prevalence among Cigar Smokers		Relative Risk for Former Exclusive Cigar Smokers			Cigar Smoking-Attributable Morbidity				
Prevalence of Disease among Former Exclusive Cigar Smokers	Prevalence of Disease among Current Exclusive Cigar Smokers	RR - Relative Risk^	RR - 1	Variance log (RR)	Cigar smoking-attributable morbidity (N * Pe * P d|ns * (RR - 1))	Variance (Var(log(Pe)) + Var(log(P d|ns)) + Var(log(RR - 1)))	Standard Error	95% Confidence Interval	
0.209 (0.164-0.263)	0.091 (0.059-0.138)	1.33	0.33	0.0289	65,184	0.0332425	0.182325	45,598	93,184
0.073 (0.047-0.111)	0.014 (0.006-0.031)	2.42	1.42	0.2916	62,025	0.2967854	0.544780	21,322	180,422
0.165 (0.123-0.219)	0.062 (0.035-0.106)	1.44	0.44	0.0400	65,771	0.0444537	0.210841	43,508	99,428
					**192,980**				
